# Complete mitogenome of *Olidiana ritcheriina* (Hemiptera: Cicadellidae) and phylogeny of Cicadellidae

**DOI:** 10.7717/peerj.8072

**Published:** 2019-11-26

**Authors:** Xian-Yi Wang, Jia-Jia Wang, Zhi-Hua Fan, Ren-Huai Dai

**Affiliations:** 1 Institute of Entomology, Guizhou University, The Provincial Key Laboratory for Agricultural Pest Management of Mountainous Region, Guizhou, Guiyang, China; 2 Jingtanggang Customs House, Tangshan, Hebei, Tangshan, China

**Keywords:** Cicadellidae, Comparative genomics, Phylogenetics, Mitogenome, Genomics

## Abstract

**Background:**

Coelidiinae, a relatively large subfamily within the family Cicadellidae, includes 129 genera and ∼1,300 species distributed worldwide. However, the mitogenomes of only two species (*Olidiana* sp. and *Taharana fasciana*) in the subfamily Coelidiinae have been assembled. Here, we report the first complete mitogenome assembly of the genus *Olidiana*.

**Methods:**

Specimens were collected from Wenxian County (Gansu Province, China) and identified on the basis of their morphology. Mitogenomes were sequenced by next-generation sequencing, following which an NGS template was generated, and this was confirmed using polymerase chain reaction and Sanger sequencing. Phylogenic trees were constructed using maximum likelihood and Bayesian analyses.

**Results:**

The mitogenome of *O. ritcheriina* was 15,166 bp long, with an A + T content of 78.0%. Compared with the mitogenome of other Cicadellidae sp., the gene order, gene content, gene size, base composition, and codon usage of protein-coding genes (PCGs) in *O. ritcheriina* were highly conserved. The standard start codon of all PCGs was ATN and stop codon was TAA or TAG; *COII*, *COIII*, and *ND4L* ended with a single T. All tRNA genes showed the typical cloverleaf secondary structure, except for *trnSer*, which did not have the dihydrouridine arm. Furthermore, the secondary structures of rRNAs (*rrnL* and *rrnS*) in *O. ritcheriina* were predicted. Overall, five domains and 42 helices were predicted for *rrnL* (domain III is absent in arthropods), and three structural domains and 27 helices were predicted for *rrnS*. Maximum likelihood and Bayesian analyses indicated that *O. ritcheriina* and other Coelidiinae members were clustered into a clade, indicating the relationships among their subfamilies; the main topology was as follows: (Deltocephalinae + ((Coelidiinae + Iassinae) + ((Typhlocybinae + Cicadellinae) + (Idiocerinae + (Treehopper + Megophthalminae))))). The phylogenetic relationships indicated that the molecular taxonomy of *O. ritcheriina* is consistent with the current morphological classification.

## Introduction

Coelidiinae is a relatively large subfamily within the Cicadellidae family, and it includes 129 genera and approximately 1,300 species (Nielson, 2015), including some species that serve as vectors of pathogens causing economically important plant diseases (Du et al., 2017; Frazier, 1975; Li & Fan, 2017; Maramorosch, Harris & Futuyma, 1981; Zhang, 1990). However, the taxonomic status of some species, on the basis of their morphology, remains controversial, and the phylogenetic relationships among major lineages of Membracoidea remain poorly understood (Dietrich et al., 2017). Moreover, knowledge regarding the taxonomic status of *Olidiana* within Cicadellidae and its phylogenetic relationship with other leafhopper genera is limited.

Complete mitogenomes provide large and diverse datasets for species delineation, and such mitogenomes have extensively been used for evolutionary studies of insects, particularly members of the orders Lepidoptera, Diptera, and Hemiptera (Salvato et al., 2008; Wang et al., 2011; Du et al., 2017; Su & Liang, 2018; Wang et al., 2018; Li et al., 2017). To date, approximately 35 species (26 complete and nine nearly complete) of the Cicadellidae mitogenome are available in GenBank. However, the mitogenomes of only two species [*Olidiana* sp. (partial genome, KY039119.1) and *Taharana fasciana* (NC_036015.1)] have previously been published for Coelidiinae, the largest subfamily of Cicadellidae.

*Olidiana* McKamey is the largest leafhopper genus in the tribe Coelidiini and it comprises 91 species. Among these, 54 species have been reported from China. However, to date, none of the characterized mitogenomes of the *Olidiana* sp. is complete; this lack of information restricts our understanding of the evolution of the Coelidiinae sp. at the genomic level. Therefore, new mitogenomic data will provide insights for determining the phylogenetic relationships and evolution of Cicadellidae in the future.

*Olidiana ritcheriina*, first described in 1990 (Zhang, 1990), is widely distributed throughout the Chinese provinces of Shaanxi, Hubei, Hunan, Guangdong, Hainan, Guangxi, Sichuan, Guizhou, and Yunnan. Therefore, a complete mitogenome of *O. ritcheriina* (GenBank accession NO.: MK738125) was sequenced to elucidate the phylogenetic status and relationships of the Coelidiinae sp.

## Materials & Methods

### Sample collection and identification

The use of the specimens collected for this study was approved. The specimens were collected from Wenxian County, Gansu Province, China (32°95′N, 104°68′E) on October 17, 2018, and identified on the basis of their morphological characteristics, as described by Zhang (1990) and Li & Fan (2017). Fresh specimens were preserved in absolute ethanol and stored at −20 °C until DNA extraction.

### DNA extraction

Genomic DNA was extracted from the whole body of adult males (after removing the abdomen and wing) using DNeasy©Tissue Kit (Qiagen, Hilden, Germany). The samples were incubated at 56 °C for 6 h for completely lysing the cells, and total genomic DNA was eluted in 100 µL of double-distilled water; the remaining steps were performed according to the manufacturer’s instructions. The extracted genomic DNA was stored at −20 °C until further use. Voucher specimens with male genitalia and DNA samples have been deposited at the Institute of Entomology, Guizhou University, Guiyang, China.

### Polymerase chain reaction (PCR) amplification and sequencing

Mitogenomes were sequenced using next-generation sequencing (Illumina HiSeq 4000 and 2 Gb raw data; Berry Genomic, Beijing, China), and two sequence fragments were reconfirmed by PCR amplification using primers (Table S1). Following this, an NGS template was generated and this was further confirmed using PCR and Sanger sequencing. PCR amplification of overlapping sequence fragments was performed using universal primers (Table S1). Two pairs of species-specific primers were designed using Primer Premier 6.0 (Premier Biosoft, Palo Alto, CA, USA) to amplify the control region (Table S1). PCR was performed using a PCR master mix (Sangon Biotech Co. Ltd., Shanghai, China), according to the manufacturer’s instructions.

### Sequence analysis

Next-generation sequences were assembled using Geneious R9 (Kearse et al., 2012). The assembled mitochondrial gene sequences were compared with the homologous sequences of *Olidiana* sp. (KY039119) and *T. fasciana* (KY886913) retrieved from GenBank and identified through BLAST searches in NCBI to confirm sequence accuracy. The sequences obtained by PCR amplification and TA cloning were assembled using SeqMan in the DNAStar software package (DNASTAR, Inc., Madison, WI, USA). The mitogenomes were annotated using the MITOS webserver (Bernt et al., 2013). Base composition and relative synonymous codon usage (RSCU) were analyzed using MEGA 6.06 (Tamura et al., 2013), and the boundaries and secondary structures of 22 tRNA genes were determined using tRNAscan-SE version 1.21 (Schattner, Brooks & Lowe, 2005) and ARWEN version 1.2 (Laslett & Canbäck, 2008). rRNA genes were identified on the basis of the locations of adjacent tRNA genes and comparisons with sequences of other Hemipterans. The secondary structures of rRNAs were inferred on the basis of models proposed for other Hemiptera (Wang, Li & Dai, 2017; Su et al., 2018). Helices were numbered according to the convention established by the Comparative RNA Web Site (Cannone et al., 2002). Strand asymmetry was calculated using the following formulas: AT skew = (A − T)/(A + T), GC skew = (G − C)/(G + C) (Perna & Kocher, 1995). Intergenic spacers and overlapping regions between genes were manually counted.

### Sequence alignment and phylogenetic analysis

The phylogenetic analysis included complete or nearly complete mitogenome sequences of 42 insect species, namely 35 leafhoppers, 5 treehoppers, 2 froghoppers (*Tettigades auropilosa* and *Cosmoscarta bispecularis*) as outgroups, and *O. ritcheriina*, which was newly sequenced (Table 1).

Each PCG and rRNA sequence was aligned using the MAFFT algorithm in Translator X (Abascal, Zardoya & Telford, 2010; Katoh, Rozewicki & Yamada, 2019) and MAFFT v7.0 online serve with the G-INS-i strategy (Castresana, 2000), respectively. Poorly aligned sites were removed using Gblocks 0.91b (Castresana, 2000) under default settings, except that the gap sites were toggled as “none”. Subsequently, the resulting 15 alignments were assessed and manually corrected using MEGA 6 (Tamura et al., 2013).

**Table 1:** Summary of the mitogenomes used in this study.

	Species	Size (bp)	A+T (%)	Accession number	Reference
Cicadellinae	*Bothrogonia ferruginea*	15,262	76.5	KU167550	Unpublished
	*Homalodisca vitripennis*	15,304	78.4	NC_006899	Unpublished
Coelidiinae	*Olidiana ritcheriina*	15,166	78.0	MK738125	**This study**
	*Olidiana* sp.^a^	15,253	78.1	KY039119	Unpublished
	*Taharana fasciana*	15,161	77.9	KY886913	Wang, Li & Dai (2017)
Deltocephalinae	*Agellus* sp.^a^	14,819	75.8	KX437738	Song, Cai & Li (2018)
	*Alobaldia tobae*^a^	16,026	77.3	KY039116	Song, Cai & Li (2017)
	*Cicadula* sp.^a^	14,929	74.1	KX437724	Song, Cai & Li (2018)
	*Drabescoides nuchalis*	15,309	75.6	NC_028154	Wu et al. (2016)
	*Exitianus indicus*^a^	16,089	75.1	KY039128	Song, Cai & Li (2017)
	*Japananus hyalinus*	15,364	76.6	NC_036298	Du et al. (2017)
	*Macrosteles quadrilineatus*	16,626	78.0	NC_034781	Mao, Yang & Bennett (2017)
	*Maiestas dorsalis*	15,352	78.7	NC_036296	Du et al. (2017)
	*Nephotettix cincticeps*	**14,805**	77.6	NC_026977	Unpublished
	*Norvellina* sp.^a^	15,594	74.5	KY039131	Song, Cai & Li (2017)
	*Orosius orientalis*^a^	15,513	72.0	KY039146	Song, Cai & Li (2017)
	*Phlogotettix sp*.	15,136	77.9	KY039135	Song, Cai & Li (2017)
	*Scaphoideus maai*	15,188	77.2	KY817243	Du, Dai & Dietrich (2017)
	*Scaphoideus nigrivalveus*	15,235	76.6	KY817244	Du, Dai & Dietrich (2017)
	*Scaphoideus varius*	15,207	75.9	KY817245	Du, Dai & Dietrich (2017)
	*Tambocerus* sp.	15,955	76.4	KT827824	Yu et al. (2017)
	*Yanocephalus yanonis*	15,623	**74.6**	NC_036131	Song, Cai & Li (2017)
Iassinae	*Trocnadella arisana*	15,131	**80.7**	NC_036480	Unpublished
Idiocerinae	*Idioscopus clypealis*	15,393	78.3	MF784430	Dai, Wang & Yang (2018)
	*Idioscopus laurifoliae*	**16,811**	79.5	MH433622	Wang et al. (2018)
	*Idioscopus sp myrica*	15,423	77.9	MH492317	Wang et al. (2018)
	*Idioscopus nitidulus*	15,287	78.6	NC_029203	Choudhary et al. (2018)
	*Populicerus populi*	16,494	77.2	MH492318	Wang et al. (2018)
Megophthalminae	*Durgades nigropicta*	15,974	78.8	NC_035684	Wang et al. (2017)
	*Japanagallia spinosa*	15,655	76.6	NC_035685	Wang et al. (2017)
Treehopper	*Darthula_hardwickii*	15,355	78.0	NC_026699	Liang, Gao & Zhao (2016)
	*Entylia carinata*	15,662	78.1	NC_033539	Mao, Yang & Bennett (2016)
	*Leptobelus gazella*	16,007	78.8	NC_023219	Zhao & Liang (2016)
	*Leptobelus* sp.	15,201	77.5	JQ910984	Li et al. (2017)
	*Tricentrus* sp.	15,419	78.5	KY039118	Unpublished
Typhlocybinae	*Empoasca onukii*	15,167	78.3	NC_037210	Liu et al. (2017)
	*Empoasca* sp.^a^	15,116	76.8	KX437737	Song, Cai & Li (2018)
	*Empoasca vitis*	15,154	78.3	NC_024838	Zhou et al. (2016)
	*Illinigina* sp.^a^	14,803	76.0	KY039129	Song, Cai & Li (2017)
	*Typhlocyba* sp.	15,223	77.1	KY039138	Song, Cai & Li (2017)
Cicadoidea	*Tettigades auropilosa*	14,944	75.0	KM000129	Unpublished
Cercopoidea	*Cosmoscarta bispecularis*	15,426	78.5	KP064511	Yang, Liu & Liang (2016)

**Notes.**

a Incomplete mitochondrial genomes.

The following five datasets were concatenated for phylogenetic analysis: (1) P123: all codon positions of 13 PCGs (10,116 bp); (2) P12: first and second codon positions of 13 PCGs (6,744 bp); (3) P123-rR: P123 and two rRNAs (11,934 bp); (4) P12-rR: P12 and two rRNAs (8,562 bp); and (5) AA: amino acid sequences of 13 PCGs (3,371 bp). The potential substitution saturation of four datasets (P123, P12, P123-rR, and P12-rR) was assessed using the index of substitution saturation (*Iss*) proposed by Xia et al. (2003) and implemented in DAMBE 5 (Xia, 2013).

Maximum likelihood (ML) analysis was performed using IQ-TREEv1.6.3 (Nguyen et al., 2014) with the best model for each partition selected under the corrected Akaike Information Criterion (AIC) using PartitionFinder2 (Table S2) (Miller, Pfeiffer & Schwartz, 2010) and evaluated using the ultrafast bootstrap approximation approach for 10,000 replicates. Bayesian (BI) analysis was performed using MrBayes 3.2 (Ronquist et al., 2012). Two independent runs with four simultaneous Markov chains (one cold and three incrementally heated at *T* = 0.2) were run for 50,000,000 generations, sampling every 100 generations under the GTR+I+G model. The best models were then selected on the basis of the corrected AIC (Nylander et al., 2004). The phylogenetic trees were visualized using FigTree 1.4.2.

## Results

### General features of the *O. ritcheriina* mitogenome

**Figure 1:**
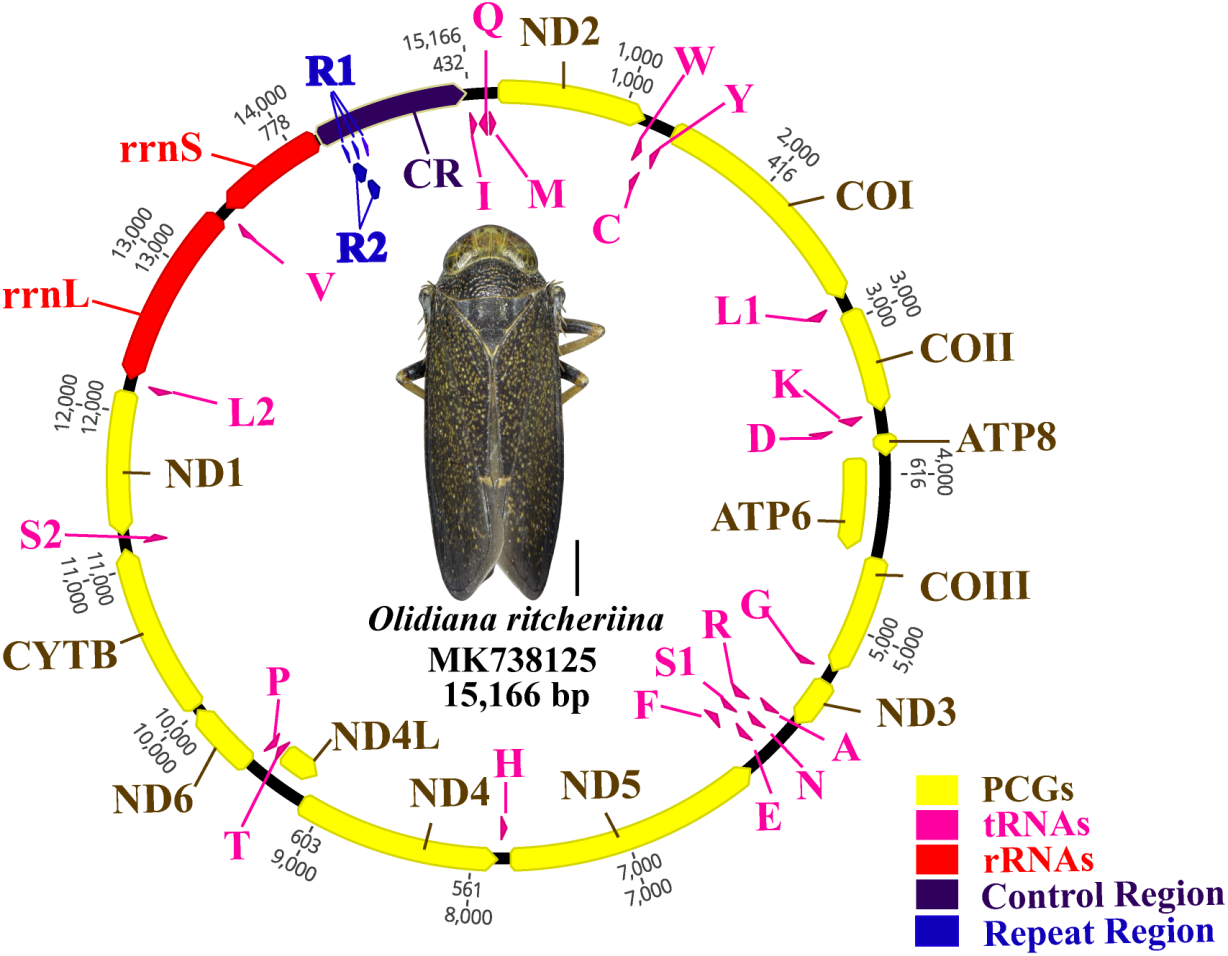
Circular map of the *Olidiana ritcheriina* mitochondrial genome. Protein coding and ribosomal genes are shown with standard abbreviations. Transfer RNA (tRNA) genes are indicated using the IUPAC-IUB single letter amino acid codes (L1, CUN; L2, UUR; S2).

The complete mitogenome of *O. ritcheriina* (MK738125) was 15,166 bp long, which is within the range of the complete mitogenomes of other Cicadellidae sp. (*Nephotettix cincticeps*, 14,805 bp and *Idioscopus laurifoliae*, 16,811 bp) (Table 1). The mitogenome comprised 37 genes (13 PCGs, 22 tRNAs, and two rRNAs) and a large A + T-rich D-loop control region (Fig. 1). The majority strand (J strand) harbored most of the genes (nine PCGs and 14 tRNAs), whereas the minority strand (N strand) harbored the remaining genes (four PCGs, two rRNAs, and eight tRNAs) (Fig. 1; Table 2). Moreover, the mitogenome of *O. ritcheriina* comprised intergenic spacers of 1 to 12 bp long at nine different loci. A total of 12 gene pairs overlapped with one another, with overlap lengths ranging from 1 to 13 bp. In addition, 16 gene pairs, including *rrnL*–*trnV* and *trnV*–*rrnS* (Table 2), were directly adjacent to one another. With a multicopy of *trnI* (AAT) located between the control region and *trnI*–*trnQ*–*trnM*, the mitogenome of *O. ritcheriina* exhibited a strong A + T bias. The A + T content of the whole genome was 78.0% (44.6% A, 33.4% T, 8.5% G, and 13.5% C) (Table 3); this percentage was between the A + T content of *Yanocephalus yanonis* (74.6%) and *Trocnadella arisana* (80.7%) (Table 1). The segment with the highest A + T content was present in the control region (83.8%); the A + T content of this segment was generally higher than that of other segments (2 rRNAs, 81.1%; 22 tRNAs, 78.6%; whole genome, 78.0%; and 13 PCGs, 77.2%) (Table 3).

**Table 2:** Composition and skewness of the *Olidiana ritcheriina* mitogenome.

Gene	Direction	Location	Size (bp)	Start	Stop	Anticodon	Intergenic nucleotides
*trnI*	J	1-62	62	–	–	GAT	
*trnQ*	N	64-130	67	–	–	TTG	1
*trnM*	J	131-196	66	–	–	CAT	0
*ND2*	J	197-1,153	957	ATT	TAA	–	0
*trnW*	J	1,152-1,213	62	–	–	TCA	2
*trnC*	N	1,201-1,262	62	–	–	GCA	−13
*trnY*	N	1,263-1,325	63	–	–	GTA	0
*COI*	J	1,338-2,873	1,536	ATG	TAA	–	12
*trnL1(UUR)*	J	2,874-2,940	67	–	–	TAA	0
*COII*	J	2,941-3,616	676	ATT	T	–	0
*trnK*	J	3,617-3,687	73	–	–	CTT	0
*trnD*	J	3,687-3,748	62	–	–	GTC	−1
*ATP8*	J	3,750-3,899	150	ATA	TAA	–	1
*ATP6*	J	3,893-4,537	645	ATG	TAA	–	−7
*COIII*	J	4,538-5,315	778	ATG	T	–	0
*trnG*	J	5,316-5,375	60	–	–	TCC	0
*ND3*	J	5,376-5,729	354	ATA	TAG	–	0
*trnA*	J	5,728-5,788	61	–	–	TGC	−2
*trnR*	J	5,788-5,852	65	–	–	TCG	−1
*trnN*	J	5,850-5,913	64	–	–	GTT	−3
*trnS1(AGN)*	J	5,913-5,974	62	–	–	GCT	−1
*trnE*	J	5,974-6,036	63	–	–	TTC	−1
*trnF*	N	6,036-6,103	68	–	–	GAA	−1
*ND5*	N	6,103-7,773	1,671	ATA	TAG	–	−1
*trnH*	N	7,774-7,834	61	–	–	GTG	0
*ND4*	N	7,834-9,150	1,317	ATG	TAG	–	−1
*ND4L*	N	9,152-9,419	278	ATG	T	–	1
*trnT*	J	9,422-9,484	63	–	–	TGT	1
*trnP*	N	9,485-9,546	62	–	–	TGG	0
*ND6*	J	9,549-10,025	477	ATA	TAA	–	2
*CYTB*	J	10,030-11,151	1,122	ATT	TAA	–	4
*trnS2(UCN)*	J	11,151-11,214	64	–	–	TGA	-1
*ND1*	N	11,216-12,146	939	ATT	TAA	–	1
*trnL2(CUN)*	N	12,147-12,214	68	–	–	TAG	0
*rrnL*	N	12,215-13,394	1,180	–	–	–	0
*trnV*	N	13,395-13,454	60	–	–	TAC	0
*rrnS*	N	13,455-14,185	731	–	–		0
A+T-rich		14,148-14,313	166	–	–	–	0

**Table 3:** Annotation of the *Olidiana ritcheriina* mitogenome.

Regions	Size	A %	G %	T %	C %	A+T %	G+C %	AT skew	GC skew
Whole genome	15,166	44.6	8.5	33.4	13.5	78.0	22.0	0.144	−0.227
PCGs	10,890	44.7	8.8	32.1	14.4	77.2	23.2	0.163	−0.250
tRNA genes	1405	43.6	9.5	34.9	11.9	78.6	21.4	0.111	−0.110
rRNA genes	1911	47.0	7.0	34.1	12.0	81.1	18.9	0.160	−0.265
Control region	981	39.9	7.8	43.9	8.4	83.8	16.2	−0.049	−0.031

Composition analysis revealed that the mitogenome of *O. ritcheriina* exhibited a positive AT (0.144) and negative GC skew (−0.227) in the whole mitogenome as well as in the 13 PCGs (AT skew: 0.163; GC skew: −0.250), 2 rRNAs (AT skew: 0.160; GC skew: −0.265), and 22 tRNAs (AT skew: 0.111; GC skew: −0.110). However, slightly negative AT (−0.049) and GC (−0.031) skews were detected in the control region (Table 3).

Comparative analysis of the base composition of every component of the mitogenomes of Coelidiinae indicated that the control regions showed the highest A + T content (81.4%–83.8%) and that all species exhibited a positive AT (0. 144 to 0.16) or negative GC (−0.227 to −0.23) skew (Wang, Li & Dai, 2017) (Table 3).

### PCGs and codon usage

The concatenated lengths of the 13 PCGs of *O. ritcheriina* were 10,116 nucleotide positions. Similar to the mitogenomes of other Cicadellidae sp., *ND5* was the largest gene (1,671 bp) and *ATP8* was the smallest gene (150 bp). Only four PCGs (*ND4*, *ND4L*, *ND5*, and *ND1*) were coded by the minority strand (N strand), whereas the other nine PCGs (*COI*, *COII*, *COIII*, *ATP8*, *ATP6*, *ND2*, *ND3*, *ND6*, and *CYTB*) were coded by the majority strand (J strand). Most PCGs exhibited the typical start codon ATN (ATA/ATT/ATG/ATC) and stop codon TAA or TAG, but *COII*, *COIII*, and *ND4L* showed an incomplete stop codon T.

**Figure 2:**
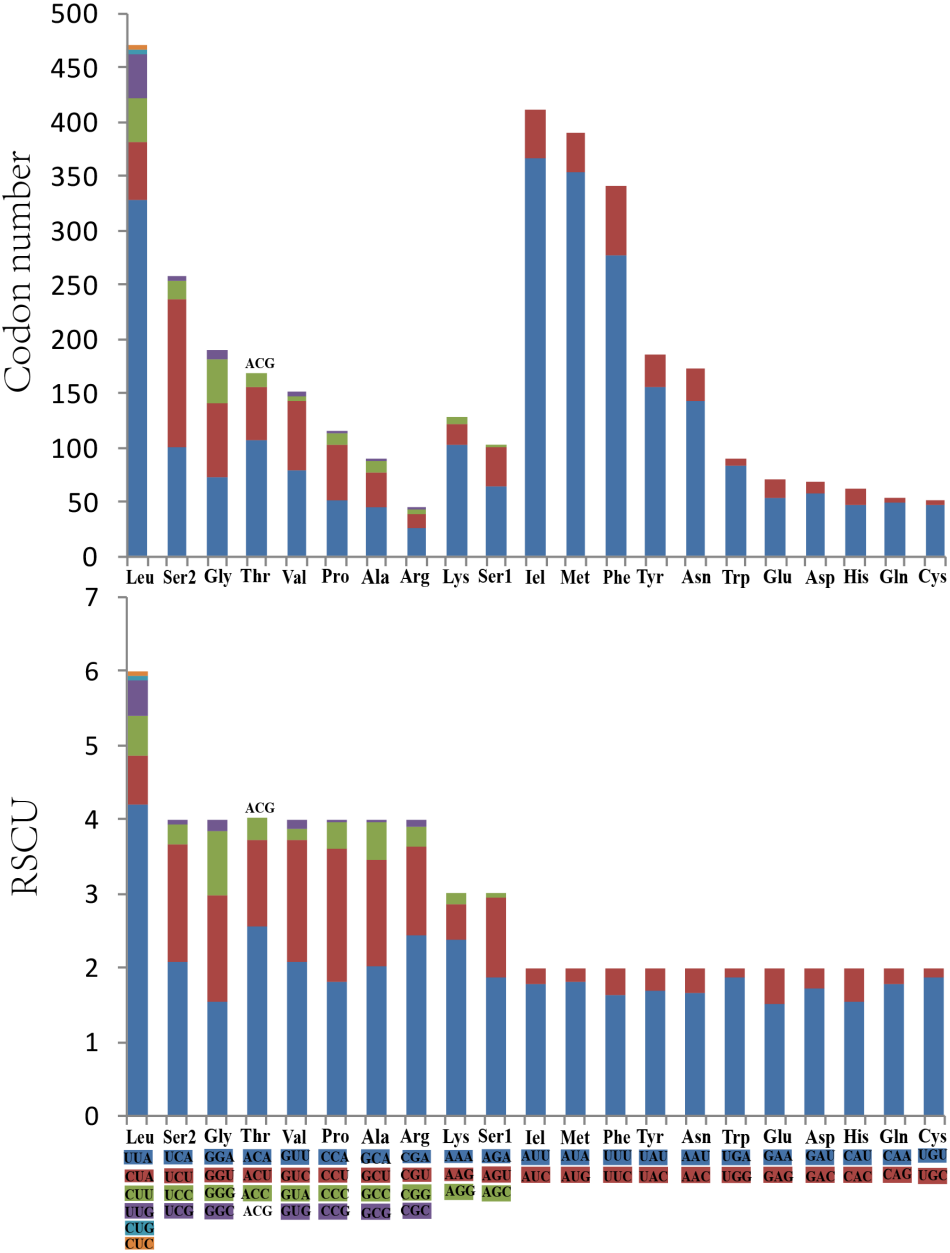
The codon number and relative synonymous codon usage (RSCU) of PCGs in *Olidiana ritcheriina* mitogenome.

Analysis of the behavior of PCG codon families revealed an extremely similar codon usage among the mitogenomes of Cicadellidae, with TTA-Leu, ATA-Met, ATT-Ile, and TTT-Phe being the four most frequently used codons (Fig. 2A). Furthermore, the RSCU of *O. ritcheriina* indicated that degenerate codons were biased to use more A/T than G/C at the third codon (Fig. 2B). Similarly, the biased usage of A + T nucleotides was reflected in the codon frequencies.

### tRNAs and rRNAs

**Figure 3:**
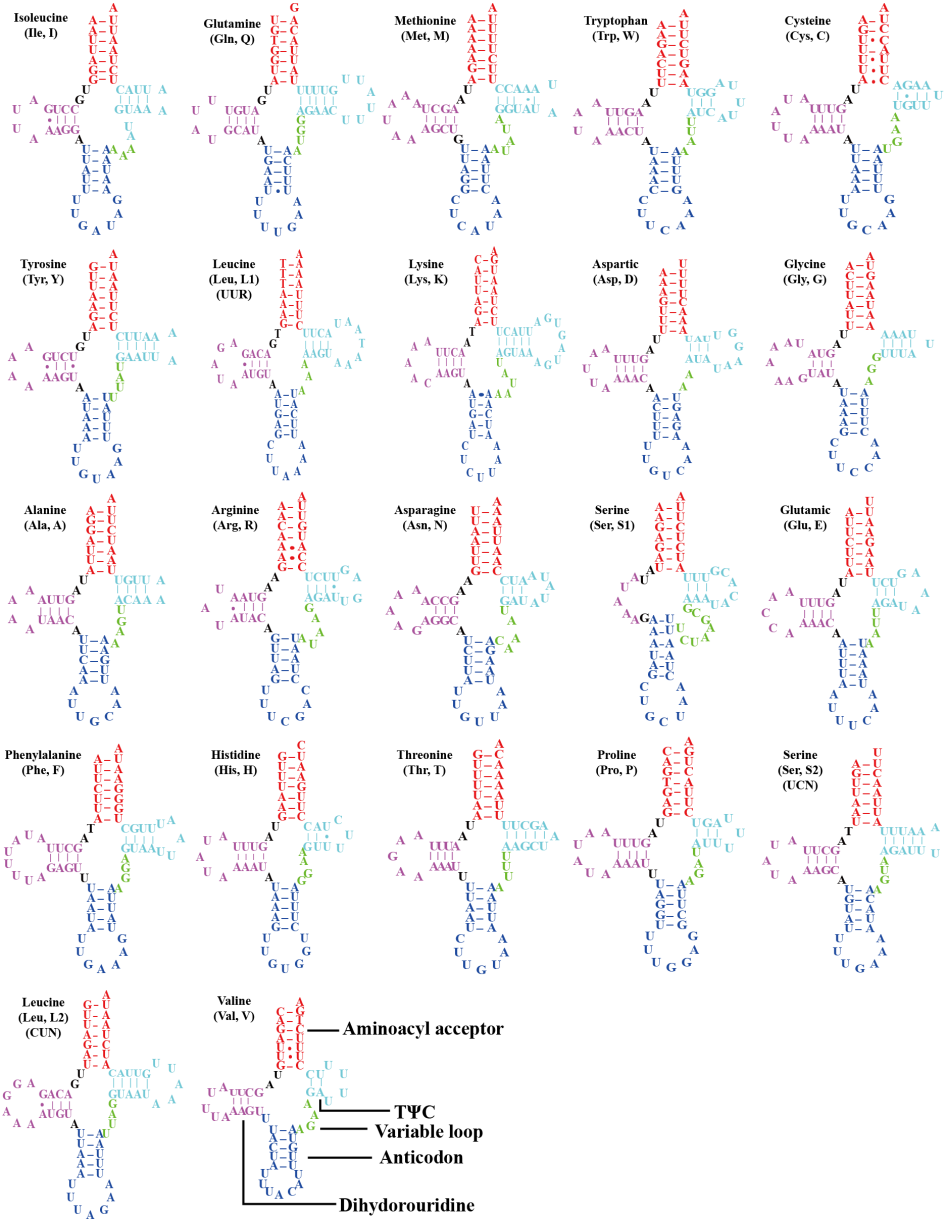
Secondary structures of tRNAs in the mitogenome of Olidiana *ritcheriina*. The dashes indicate Watson-Crick bonds and GU pairs, solid dots indicate mismatches.

All the 22 typical tRNA genes were present in the mitogenome of *O. ritcheriina*, and their lengths ranged between 61 (*trnA* and *trnH*) and 71 bp (*trnK*). All tRNAs were identified using tRNAscan-SE (Schattner, Brooks & Lowe, 2005) and ARWEN (Laslett & Canbäck, 2008). Among these, 14 were located on the J strand and eight on the N strand. All tRNAs exhibited the typical cloverleaf secondary structure, with the exception of *trnS1* (AGN) in which the dihydrouridine arm formed a loop (Fig. 3). Abascal et al. (2006) and Abascal, Posada & Zardoya (2012) have shown that the invertebrate mitochondrial genetic code even shifts within the Hemiptera, with *Triatoma* (Cimicomorpha), *Homalodisca* (Cicadellidae), and *Philaenus* (Cercopoidea) using the AGG codon that was translated as Lys instead of Ser; accordingly, our tRNA analysis shows that the AGG codon in *O. ritcheriina* was translated as Lys instead of Ser.

**Figure 4:**
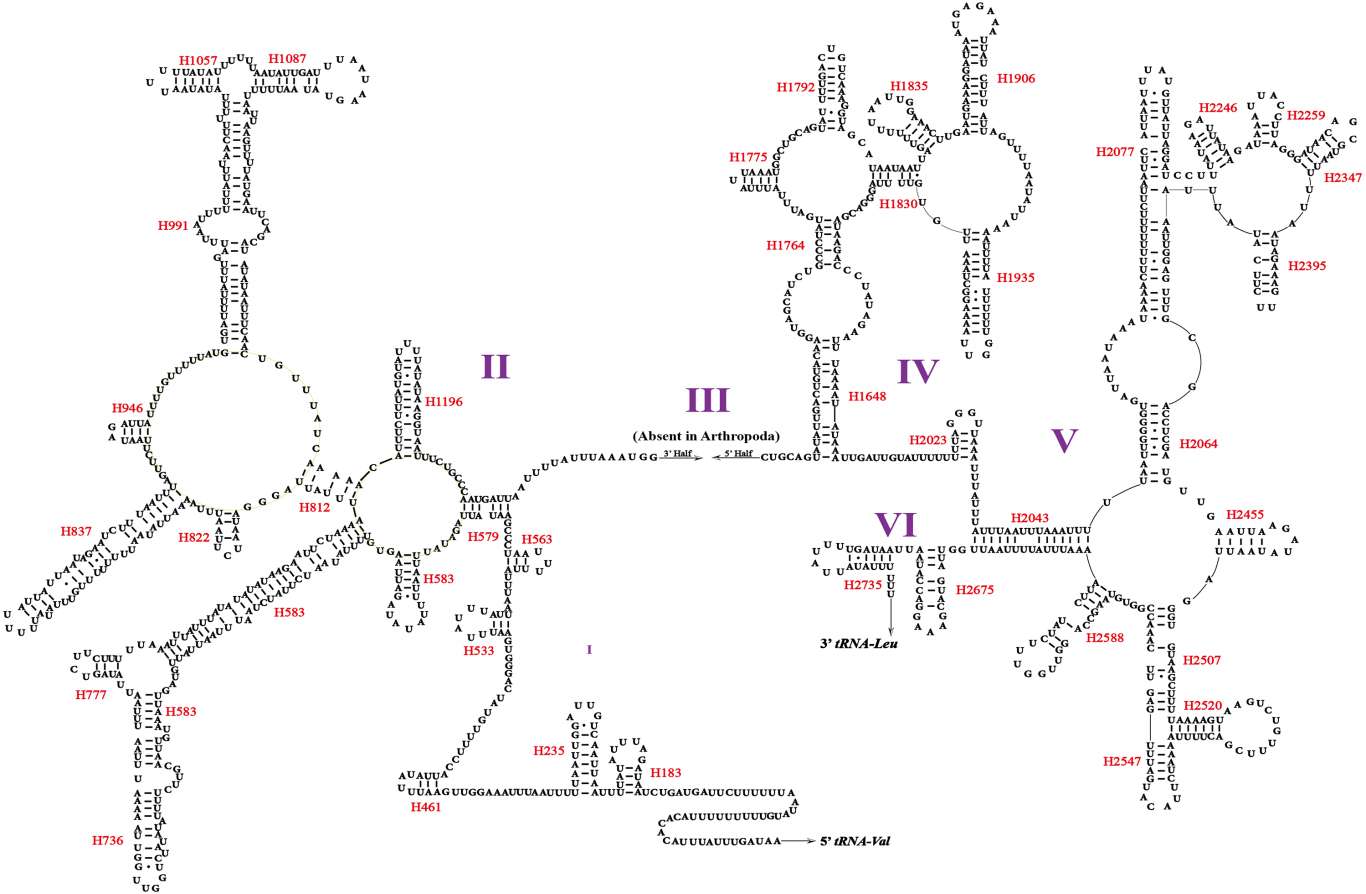
Predicted secondary structure of the *rrnL.* in the mitogenome of *Olidiana ritcheriina*. Roman numerals indicate the conserved domain structure. Watson-Crick pairs are joined by dashes, hereas GU pairs are connected by dots.

Two rRNA genes (*rrnL* and *rrnS*) in the mitogenomes of Cicadellidae were highly conserved. The putative lengths of the *O. ritcheriina* genes *rrnL* and *rrnS* were 1,180 bp between *trnL2* and *trnV* and 731 bp between *trnV* and the control region, respectively (Tables 2 and 3). In the mitogenomes of Coelidiinae, the length of *rrnL* ranged from 1,178 (*Olidiana* sp.) to 1,192 bp (*T. fasciana*) and that of *rrnS* ranged from 729 (*Olidiana* sp.) to 775 bp (*T. fasciana*). The secondary structure of the *O. ritcheriina* gene *rrnL* comprised five domains (I, II, IV, V, and VI; domain III is absent in arthropods) and 42 helices (Fig. 4). Multiple alignment of the Coelidiinae gene *rrnL* extended over 1,180 positions and comprised 1,016 conserved (86.10%) and 164 variable (13.90%) sites. Domains IV and V were structurally more conserved than the other domains.

**Figure 5:**
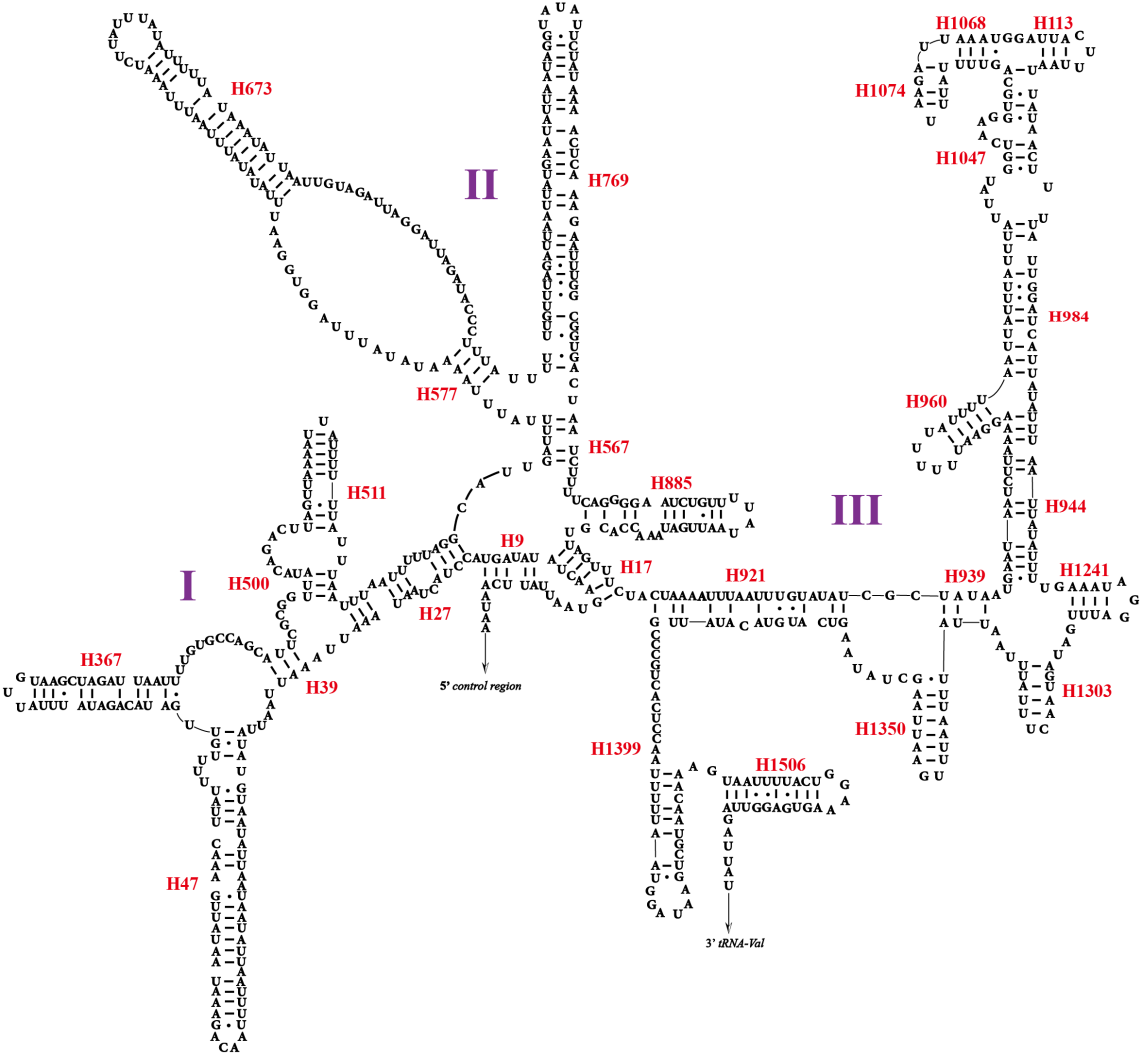
Predicted secondary structure of the *rrnS.* in the mitogenome of *Olidiana ritcheriina*. Roman numerals indicate the conserved domain structure. Watson-Crick pairs are joined by dashes, hereas GU pairs are connected by dots.

The secondary structure of *rrnS* comprised three structural domains and 27 helices (Fig. 5). Multiple alignments of the Coelidiinae gene *rrnS* extended over 730 positions and comprised 586 conserved (80.23%) and 164 variable (19.73%) sites. Domain III was structurally more conserved than domains I and II.

These rRNA secondary structures can be useful for the precise alignment of sequences for phylogenetic studies (Rijk & Wachter, 1997). Nevertheless, additional details regarding such rRNA structures should be accumulated in future studies.

### Control region

**Figure 6:**
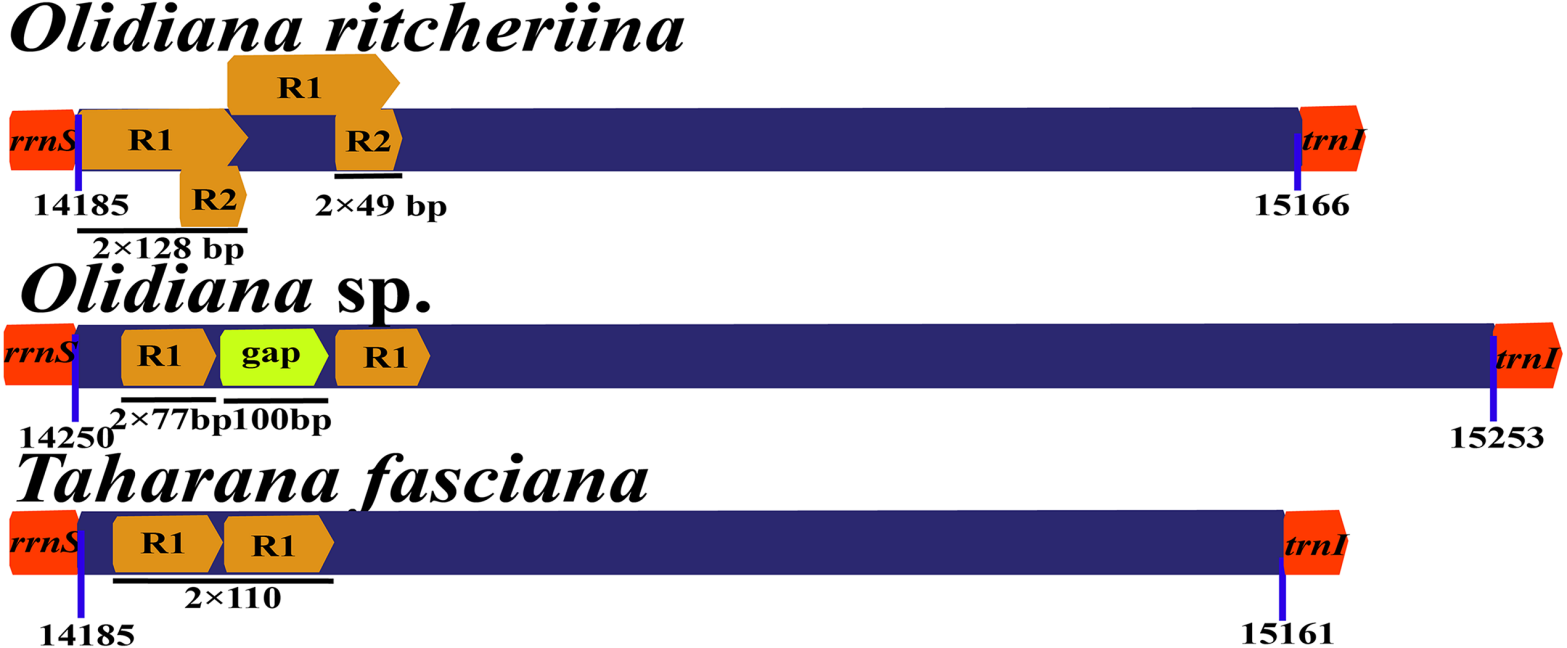
Organization of the control region structure in the mitochondrial genomes of *Olidiana ritcheriina*. R, repeat unit.

The control regions (A + T-rich regions) in the mitogenomes of Coelidiinae were not highly conserved, with lengths ranging between 915 (*T. fasciana*) and 1,069 bp (*Olidiana* sp.) and A + T content ranging between 77.9% (*T. fasciana*) and 78.1% (*Olidiana* sp.) (Table 1). The length of the control region of *O. ritcheriina* was 981 bp, with a high A + T content (83.8%) and two repeats: R1 (2 × 49 bp) and R2 (2 × 128 bp) (Fig. 6A). However, the control regions of *T. fasciana* and *Olidiana* sp. comprised a single repeat (Figs. 6B–6C). In addition, the control region of the *O. ritcheriina* showed slightly negative AT (−0.049) and GC (−0.031) skews (Table 3).

### Phylogenetic relationship

Phylogenetic trees were constructed on the basis of five concatenated nucleotide sequence datasets from 40 available mitogenomes of Membracoidea, with two species considered outgroups [Cicadoidea (*T. auropilosa*) and Cercopoidea (*C. bispecularis*)]. Saturation analysis addresses the issue on whether some positions or partitions of a dataset are saturated and to test whether these sites can be used for further phylogenetic analysis. These phylogenetic trees showed uncorrected pairwise divergence in transitions (s) and transversions (v) against divergences calculated with the GTR model, and none of the four candidate nucleotide sequence datasets (Fig. S1A: P123; Fig. S1B: P12; Fig. S1C: P123-rR; Fig. S1D: P12-rR) had reached saturation (all *Iss* <*Iss*. *cSym* or *Iss*. *cAsym*, *p* = 0.0000) (Table 4; Fig. S1), thereby suggesting that the concatenated data is suitable for phylogenetic analysis.

**Figure 7:**
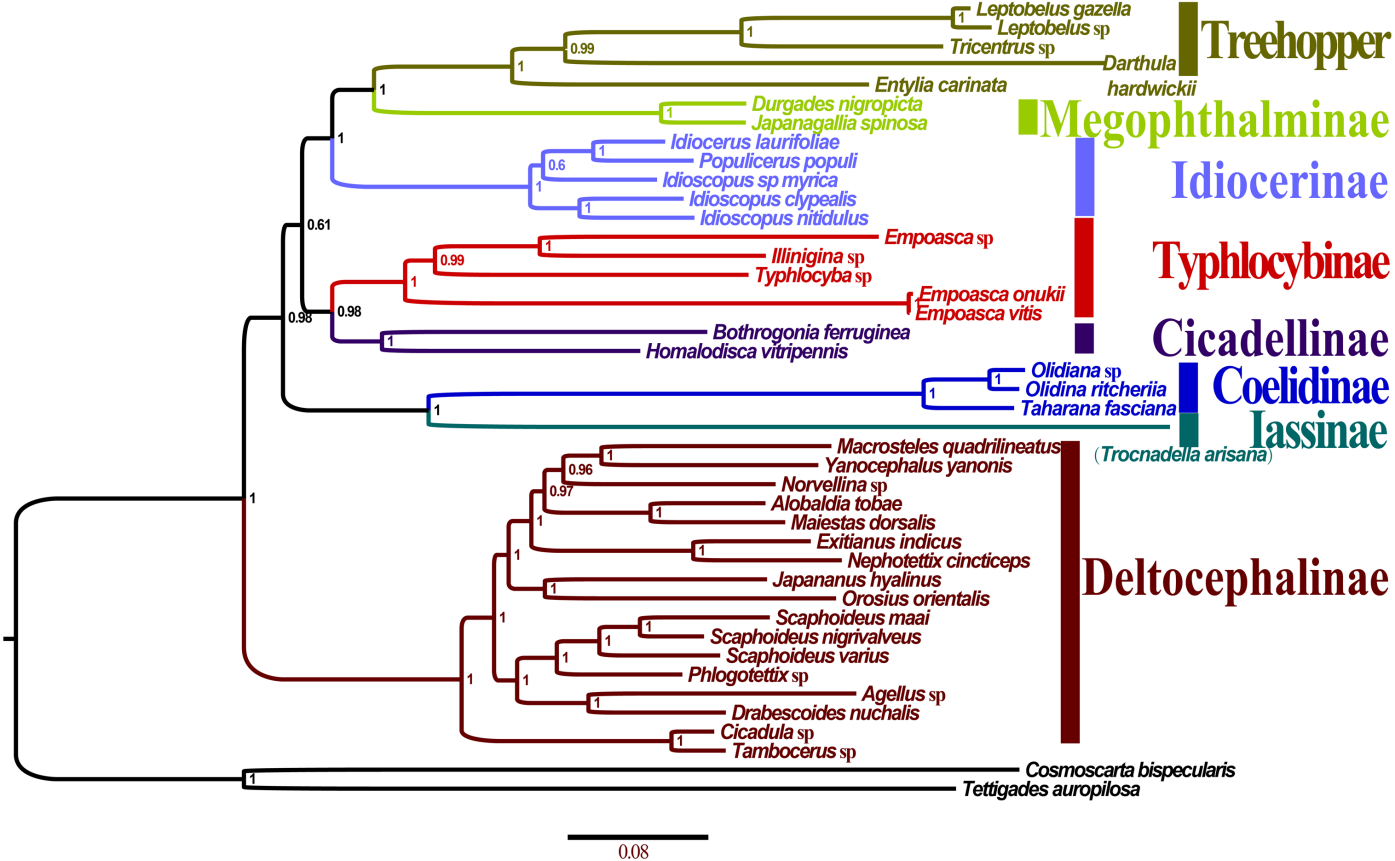
Phylogenetic trees of *Olidiana ritcheriina.* inferred based on the first and second codon positions of 13 PCGs using GTR+I+G model in MrBayes.

All the 10 trees are presented in Fig. 7 and Fig. S2A–F. Almost all nodes received high support (posterior probability, PP >0.88) in BI analyses, whereas a few nodes received only moderate or low support in ML analyses of some datasets (bootstrap support, BS <75). Monophyly at the subfamily level within Membracoidea was strongly supported in all the trees. Membracidae as a sister group to Cicadellidae was well supported by all the results (*PP* > 0.94, BS = 100). Within Cicadellidae, the 37 species sampled in this study represent seven subfamilies and the main topology was as follows: (Deltocephalinae + ((Coelidiinae + Iassinae) + ((Typhlocybinae + Cicadellinae) + (Idiocerinae + (Treehopper + Megophthalminae))))) (Fig. 7). The results of BI and ML analyses generated results that are consistent with those of previous phylogenetic studies on the basis of combined morphological and molecular data (Dietrich et al., 2001; Dietrich et al., 2017; Cryan et al., 2000; Cryan & Urban, 2012; Krishnankutty, 2013; Wang, Dietrich & Zhang, 2017).

**Table 4:** Substitution saturation tests for the four dataset.

Dataset	Observed *Iss*	*Iss.cSym*^a^	*Psym*^b^	*Iss.cAsym*^c^	*Pasym*^d^	Dataset	Observed *Iss*	*Iss.cSym*^a^	*Psym*^b^	*Iss.cAsym*^c^
P123	0.419	0.817	0.0000	0.571	0.0000	P123-rR	0.420	0.818	0.0000	0.572
P12	0.296	0.814	0.0000	0.570	0.0000	P12-rR	0.320	0.816	0.0000	0.571

**Notes.**

NumOUT = 32

a Critical values assuming a symmetrical tree.

b Signifcant difference between *Iss* and *Iss.cSym* (two-tailed test).

c Critical values assuming an extreme asymmetrical tree.

d Signifcant difference between *Iss* and *Iss.cAsym* (two-tailed *t*-test).

## Discussion

The phylogenetic relationships inferred according to the five datasets showed slightly different topologies. In the BI-P123-rR/ML-P12-rR/ML-P123-rR analysis, the main topology was as follows: (Typhlocybinae + (Cicadellinae + (Deltocephalinae + ((Coelidiinae + Iassinae) + (Idiocerinae + (Treehopper + Megophthalminae)))))) (Fig. S2C). This topology is consistent with that reported in a previous study (Du et al., 2017) based on BI analysis of amino acid sequences. However, in some other studies (Du et al., 2017; Wang et al., 2018), the main topology has been reported to be different, i.e., (Deltocephalinae + (Typhlocybinae + (Cicadellinae + ((Coelidiinae + Iassinae) + (Idiocerinae + (Treehopper + Megophthalminae)))))) (Fig. S2D). This difference can be mainly attributed to the unstable positions of Deltocephalinae, Typhlocybinae, and Coelidiinae and Iassinae (Fig. 7).

In Membracoidea, three clades exhibited a stable sister relationship as shown in all trees in the present analysis: Idiocerinae + (Typhlocybinae + Cicadellinae); Coelidiinae + Iassinae; and Treehopper + Megophthalminae. This result is consistent with that reported in some previous studies (Dietrich et al., 2001; Dietrich et al., 2017; Krishnankutty, 2013; Wang et al., 2017; Wang et al., 2018). Coelidiinae was the most closely related to Iassinae in the present study according to the BI (*PP* = 1.00) and ML (BS = 100) trees, which were the same as those reported in previous studies (Wang et al., 2017; Wang et al., 2018). Within Coelidiinae, the three species sampled in the present study represent *Olidiana* and *Taharana*. The inferred relationships (*Taharana fascianus* + (*Olidiana* sp. + *Olidiana ritcheriina*)) were well supported by all BI (PP = 1.00) and ML (BS = 100) trees. The third codon position shows higher saturation than the first and second codon positions (Wei et al., 2010; Song, Liang & Bu, 2012) (Table S2). Nevertheless, in our phylogenetic results, tree topologies were consistent regardless of whether the third codon position was excluded; however, this exclusion slightly increased support for some nodes in ML analyses (ML-13PCGs12/ML-13PCGs and ML-13PCGs12-2RNA/ML-13PCGs-2RNA) (Figs. S2C and S2F). The results of the present study are consistent with those of a previous phylogenetic study (Du et al., 2017).

## Conclusions

We sequenced the mitogenome of *O*. *ritcheriina* from Coelidiinae and presented their structure and sequence characteristics. Consistent with previous observations related to Membracoidea, the mitogenome of *O. ritcheriina* was highly conserved in terms of gene content, gene size, gene order, base composition, PCG codon usage, as well as tRNA and rRNA secondary structures.

Furthermore, the phylogeny of Membracoidea was inferred with all 40 complete mitogenomes, namely, 35 Cicadellidae and five Treehopper. The overall phylogenetic structure of Membracoidea is consistent with that reported in previous studies. Coelidiinae was grouped with a clade comprising Iassinae. The mitogenomic information of *O. ritcheriina* can be useful for future studies aimed at exploring the mitogenomic diversity of insects and evolution of related insect lineages.

The lack of complete mitogenomes of Coelidiinae sp. has restricted the understanding of the evolution of this group at the genome level. Therefore, further studies are required to elucidate the phylogenetic status of species belonging to this group and their relationships. In this context, the addition of more taxa and genes to the leafhopper mitogenomic dataset may contribute to the determination of the relationships shared among major leafhopper lineages.

##  Supplemental Information

10.7717/peerj.8072/supp-1 Figure S1 Saturation plots for, A: P123; B: P12; C: P123-rR; C: P12-rR. The plot showed uncorrected pairwise divergences in transitions (s) and transversions (v) against divergences calculated using the GTR model. Blue: transitions; Green: transversions

10.7717/peerj.8072/supp-2 Figure S2 Phylogenetic trees of Cicadellidae inferred from BI analysis and ML analysis methods based on five datasets

10.7717/peerj.8072/supp-3 Table S1 Primers used for mitogenome analysis

10.7717/peerj.8072/supp-4 Table S2 Partition strategies and evolutionary models used in ML analysis

10.7717/peerj.8072/supp-5 Supplemental Information 5 Sequence of mitogenome of *Olidiana ritcheriina*

10.7717/peerj.8072/supp-6 Supplemental Information 6 Sequence of mitogenome of *Olidiana ritcheriina*

## Additional Information and Declarations

### Field Study Permissions

The following information was supplied relating to field study approvals (i.e., approving body and any reference numbers): The Institute of Entomology of Guizhou University approved field collection.

### DNA Deposition

The following information was supplied regarding the deposition of DNA sequences: The species sequences are available at GenBank: MK738125.

### Data Availability

The following information was supplied regarding data availability: The mitogenome sequence of *Olidiana ritcheriina* is available as a Supplemental File.

## References

[ref-1] Abascal F, Posada D, Knight RD, Zardoya R (2006). Parallel evolution of the genetic code in arthropod mitochondrial genomes. PLOS Biology.

[ref-2] Abascal F, Posada D, Zardoya R (2012). The evolution of the mitochondrial genetic code in arthropods revisited. Mitochondrial DNA.

[ref-3] Abascal F, Zardoya R, Telford JM (2010). TranslatorX: multiple alignment of nucleotide sequences guided by amino acid translations. Nucleic Acids Research.

[ref-4] Bernt M, Donath A, Juehling F, Externbrink F, Florentz C, Fritzsch G, Puetz J, Middendorf M, Stadler PF (2013). MITOS: improved de novo metazoan mitochondrial genome annotation. Molecular Phylogenetics and Evolution.

[ref-5] Cannone JJ, Subramanian S, Schnare MN, Collett JR, D’Souza LM, Du Y, Feng B, Lin N, Madabusi LV, Müller KM, Pande N, Shang Z, Yu N, Gutell RR (2002). The Comparative RNA Web (CRW) site: an online database of comparative sequence and structure information for ribosomal, intron, and other RNAs. BMC Bioinformatics.

[ref-6] Castresana J (2000). Selection of conserved blocks from multiple alignments for their use in phylogenetic analysis. Molecular Biology and Evolution.

[ref-7] Choudhary JS, Naaz N, Das B, Bhatt BP, Prabhakar CS (2018). Complete mitochondrial genome of Idioscopus nitidulus (Hemiptera: Cicadellidae). Mitochondrial DNA B.

[ref-8] Cryan JR, Urban J (2012). Higher level phylogeny of the insect order Hemiptera: is Auchenorrhyncha really paraphyletic?. Systematic Entomology.

[ref-9] Cryan JR, Wiegmann BM, Deitz LL, Dietrich CH (2000). Phylogeny of the treehoppers (Insecta: Hemiptera: Membracidae): evidence from two nuclear genes. Molecular Phylogenetics and Evolution.

[ref-10] Dai RH, Wang JJ, Yang MF (2018). The complete mitochondrial genome of the leafhopper Idioscopus clypealis (Hemiptera: Cicadellidae: Coelidiinae). Mitochondrial DNA B.

[ref-11] Dietrich CH, Allen JM, Lemmon AR, Lemmon EM, Takiya DM, Evangelista O, Walden KKO, Grady PGS, Johnson KP (2017). Anchored hybrid enrichment-based phylogenomics of leafhoppers and treehoppers (Hemiptera: Cicadomorpha: Membracoidea). Insect Systematics and Diversity.

[ref-12] Dietrich CH, Rakitov RA, Holmes JL, Black IVWC (2001). Phylogeny of the major lineages of Membracoidea (Insecta: Hemiptera: Cicadomorpha) based on 28S rDNA sequences. Molecular Phylogenetics and Evolution.

[ref-13] Du Y, Dai W, Dietrich CH (2017). Mitochondrial genomic variation and phylogenetic relationships of three groups in the genus Scaphoideus (Hemiptera: Cicadellidae: Deltocephalinae). Scientific Reports.

[ref-14] Du Y, Zhang C, Dietrich CH, Zhang Y, Dai W (2017). Characterization of the complete mitochondrial genomes of *Maiestas dorsalis* and *Japananus hyalinus* (Hemiptera: Cicadellidae) and comparison with other Membracoidea. Scientific Reports.

[ref-15] Frazier NW (1975). Possible transmission of strawberry pallidosis by the leafhopper Coelidia olitoria. Plant Disease Reporter.

[ref-16] Katoh K, Rozewicki J, Yamada KD (2019). MAFFT online service: multiple sequence alignment, interactive sequence choice and visualization. Briefings in Bioinformatics.

[ref-17] Kearse M, Moir R, Wilson A, Stones-Havas S, Cheung M, Sturrock S, Buxton S, Cooper A, Markowitz S, Duran C (2012). Geneious basic: an integrated and extendable desktop software platform for the organization and analysis of sequence data. Bioinformatics.

[ref-18] Krishnankutty S (2013). Systematics and biogeography of leafhoppers in Madagascar. Dissertations & Theses—Gradworks.

[ref-19] Laslett D, Canbäck B (2008). ARWEN: a program to detect tRNA genes in metazoan mitochondrial nucleotide sequences. Bioinformatics.

[ref-20] Li H, Leavengood JM, Chapman EG, Burkhardt D, Song F, Jiang P, Liu J, Zhou X, Cai W (2017). Mitochondrial phylogenomics of Hemiptera reveals adaptive innovations driving the diversification of true bugs. Proceedings of the Royal Society B: Biological Sciences.

[ref-21] Li ZZ, Fan ZH (2017). Coelidiinae (*Hemiptera:Cicadellidae*) from China.

[ref-22] Liang AP, Gao J, Zhao X (2016). Characterization of the complete mitochondrial genome of the treehopper Darthula hardwickii (Hemiptera: Aetalionidae). DNA Sequence.

[ref-23] Liu JH, Sun CY, Long J, Guo JJ (2017). Complete mitogenome of tea green leafhopper, Empoasca onukii (Hemiptera: Cicadellidae) from Anshun, Guizhou Province in China. Mitochondrial DNA B.

[ref-24] Mao M, Yang XS, Bennett G (2016). The complete mitochondrial genome of *Entylia carinata* (Hemiptera: Membracidae). Mitochondrial DNA B.

[ref-25] Mao M, Yang XS, Bennett G (2017). The complete mitochondrial genome of *Macrosteles quadrilineatus* (Hemiptera: Cicadellidae). Mitochondrial DNA B.

[ref-26] Maramorosch K, Harris KF, Futuyma DJ (1981). Leafhopper vectors and plant disease agents, Netherlands. Journal of Plant Pathology.

[ref-27] Miller MA, Pfeiffer W, Schwartz T (2010).

[ref-28] Nguyen LT, Schmidt HA, Von Haeseler A, Minh BQ (2014). IQ-TREE: a fast and effective stochastic algorithm for estimating maximum-likelihood phylogenies. Molecular Biology and Evolution.

[ref-29] Nielson MW (2015). A revision of the tribe Coelidiini of the Oriental, Palearctic and Australian biogeographical regions (Hemiptera: Cicadellidae: Coelidiinae). Insecta Mundi.

[ref-30] Nylander JA, Ronquist F, Huelsenbeck JP, Nieves-Aldrey J (2004). Bayesian phylogenetic analysis of combined data. Systematic Biology.

[ref-31] Perna NT, Kocher TD (1995). Patterns of nucleotide composition at fourfold degenerate sites of animal mitochondrial genomes. Journal of Molecular Evolution.

[ref-32] Rijk PD, Wachter RD (1997). Rnaviz, a program for the visualisation of RNA secondary structure. Nucleic Acids Research.

[ref-33] Ronquist F, Teslenko M, Van MP, Ayres DL, Darling A, Höhna S, Larget B, Liu L, Suchard MA, Huelsenbeck JP (2012). MrBayes 3.2: efficient bayesian phylogenetic inference and model choice across a large model space. Systematic Biology.

[ref-34] Salvato P, Simonato M, Battisti A, Negrisolo E (2008). The complete mitochondrial genome of the bag-shelter moth *Ochrogaster lunifer* (lepidoptera, notodontidae). BMC Genomics.

[ref-35] Schattner P, Brooks AN, Lowe TM (2005). The tRNAscan-SE, snoscan and snoGPS web servers for the detection of tRNAs and snoRNAs. Nucleic Acids Research.

[ref-36] Song N, Cai WZ, Li H (2017). Deep-level phylogeny of Cicadomorpha inferred from mitochondrial genomes sequenced by NGS. Scientific Reports.

[ref-37] Song N, Cai WZ, Li H (2018). Insufficient power of mitogenomic data in resolving the auchenorrhynchan monophyly. Zoological Journal of the Linnean Society.

[ref-38] Song N, Liang AP, Bu CP (2012). A molecular phylogeny of hemiptera inferred from mitochondrial genome sequences. PLOS ONE.

[ref-39] Su TJ, He B, Li K, Liang AP (2018). Comparative analysis of the mitochondrial genomes of oriental spittlebug trible cosmoscartini: insights into the relationships among closely related taxa. BMC Genomics.

[ref-40] Su TJ, Liang AP (2018). Characterization of the complete mitochondrial genome of *Phymatostetha huangshanensis* (Hemiptera: Cercopidae) and phylogenetic analysis. International Journal of Biological Macromolecules.

[ref-41] Tamura K, Stecher G, Peterson D, Filipski A, Kumar S (2013). MEGA6: molecular evolutionary genetics analysis version 6.0. Molecular Biology and Evolution.

[ref-42] Wang JJ, Dai RH, Li H, Zhan HP (2017). Characterization of the complete mitochondrial genome of *Japanagallia spinosa* and *Durgades nigropicta* (Hemiptera: Cicadellidae: Megophthalminae). Biochemical Systematics & Ecology.

[ref-43] Wang Y, Dietrich CH, Zhang Y (2017). Phylogeny and historical biogeography of leafhopper subfamily Evacanthinae (Hemiptera: Cicadellidae) based on morphological and molecular data. Scientific Reports.

[ref-44] Wang S, Lei Z, Wang H, Dong B, Ren B (2011). The complete mitochondrial genome of the *leafminerliriomyza trifolii* (diptera: agromyzidae). Molecular Biology Reports.

[ref-45] Wang JJ, Li H, Dai RH (2017). Complete mitochondrial genome of *Taharana fasciana* (Insecta, Hemiptera: Cicadellidae) and comparison with other Cicadellidae insects. Genetica.

[ref-46] Wang JJ, Yang MF, Dai RH, Li H, Wang XY (2018). Characterization and phylogenetic implications of the complete mitochondrial genome of idiocerinae (Hemiptera: Cicadellidae). International Journal of Biological Macromolecules.

[ref-47] Wei SJ, Shi M, Sharkey MJ, Achterberg CV, Chen XX (2010). Comparative mitogenomics of braconidae (insecta: hymenoptera) and the phylogenetic utility of mitochondrial genomes with special reference to holometabolous insects. BMC Genomics.

[ref-48] Wu YF, Dai RH, Zhan HP, Qu L (2016). Complete mitochondrial genome of *Drabescoides nuchalis* (Hemiptera: Cicadellidae). Mitochondrial DNA A.

[ref-49] Xia X (2013). DAMBE5: a comprehensive software package for data analysis in molecular biology and evolution. Molecular Biology and Evolution.

[ref-50] Xia X, Xie Z, Salemi M, Chen L, Wang Y (2003). An index of substitution saturation and its application. Molecular Phylogenetics and Evolution.

[ref-51] Yang H, Liu J, Liang AP (2016). The complete mitochondrial genome of *Cosmoscarata bispecularis* (Hemiptera, Cicadomorpha, Cercopoidea, Cercopidae). Mitochondrial DNA A.

[ref-52] Yu PF, Wang MX, Cui L, Chen XX, Han BY (2017). The complete mitochondrial genome of *Tambocerus* sp. (Hemiptera: Cicadellidae). Mitochondrial DNA A.

[ref-53] Zhang YL (1990). A taxonomic study of Chinese Cicadellidae (Homoptera).

[ref-54] Zhao X, Liang AP (2016). Complete DNA sequence of the mitochondrial genome of the treehopper *Leptobelus gazella* (Membracoidea: Hemiptera). Mitochondrial DNA A.

[ref-55] Zhou N, Wang M, Cui L, Chen XX, Han BY (2016). Complete mitochondrial genome of Empoasca vitis (Hemiptera: Cicadellidae). Mitochondrial DNA A.

