# Supplementary figures and images for: Complete mitogenome of Olidiana ritcheriina (Hemiptera: Cicadellidae) and phylogeny of Cicadellidae
Source: PeerJ. 2019 Nov 26;7:e8072. doi: 10.7717/peerj.8072 (PMC6883956; doi:10.7717/peerj.8072)

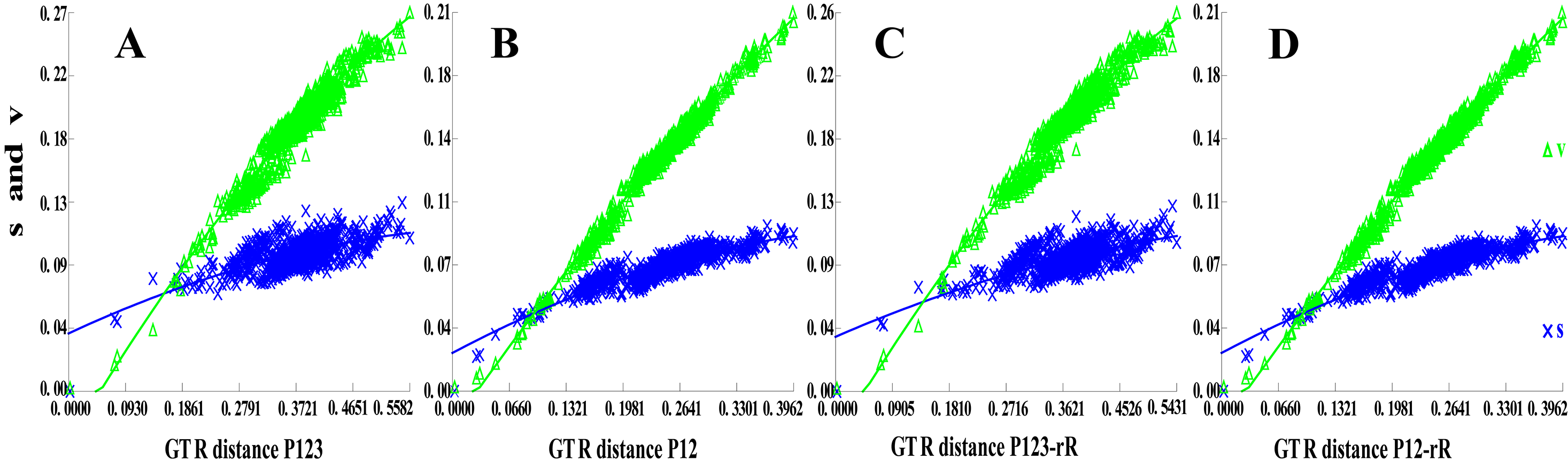

Supplement: Figure S1 [file peerj-07-8072-s001.png]

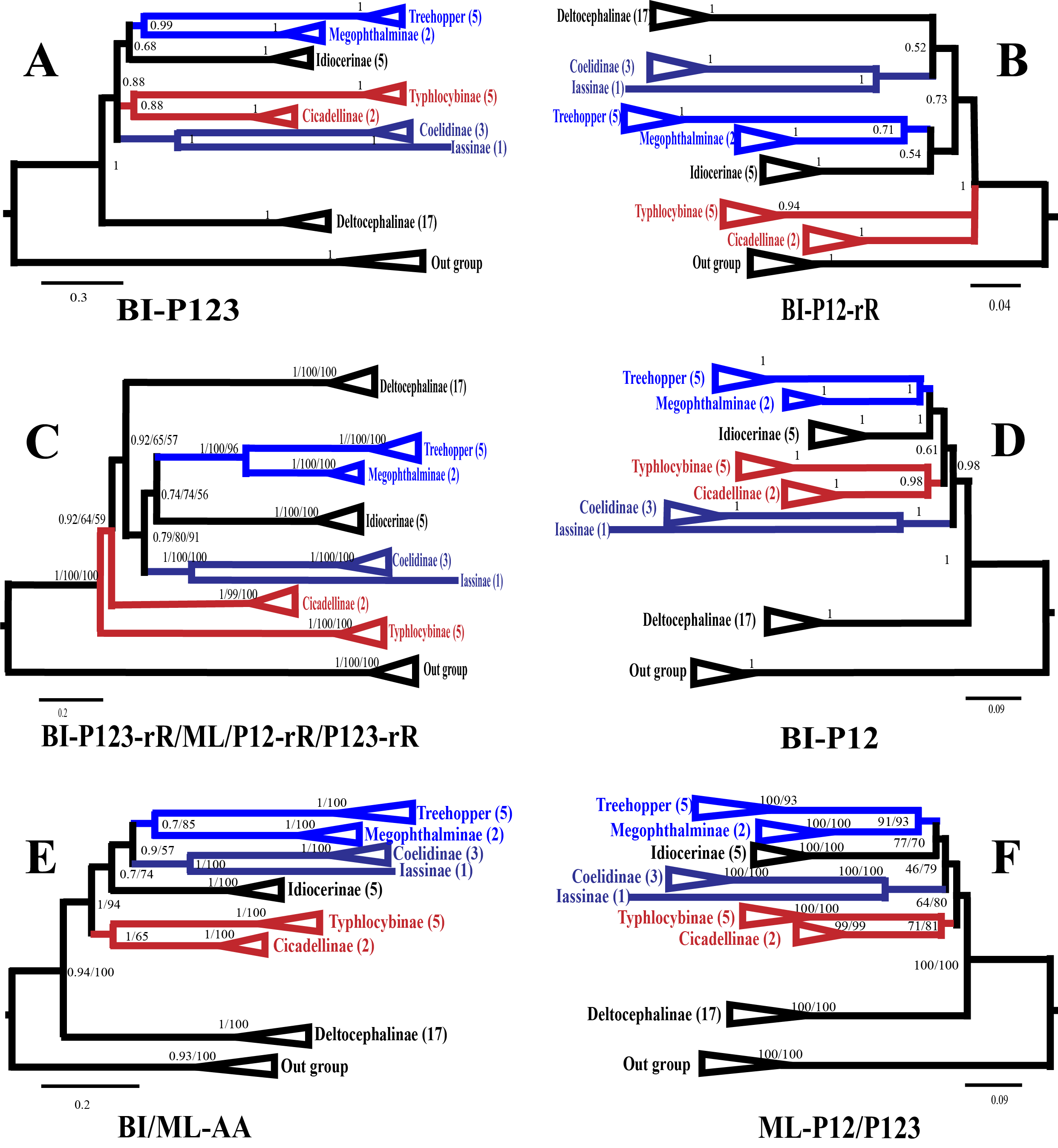

Supplement: Figure S2 [file peerj-07-8072-s002.png]
