# Supplementary material for: Complete mitogenome of Olidiana ritcheriina (Hemiptera: Cicadellidae) and phylogeny of Cicadellidae
Source: PeerJ. 2019 Nov 26;7:e8072. doi: 10.7717/peerj.8072 (PMC6883956; doi:10.7717/peerj.8072)
Supplement: Table S1 [file peerj-07-8072-s003.docx]

| Primers (5′-3′) | Annealing temperature | References |
| --- | --- | --- |
| ID-COIF: GGTCAACAAATCATAAAGATATTGG | 50 °C | (Wu et al., 20016) |
| ID-COIR: TAAACTTCAGGGTGACCAAAAAATCA | 50 °C | (Wu et al., 20016) |
| ID-12SF1: AGTCACCGCCAAATTCTT | 48 °C | Designed |
| ID-12SR1: TCCTTTAATCAGGCACCTAA | 48 °C | Designed |
| ID-12SF2: AGATTAGTCACCGCCAAAT | 48 °C | Designed |
| ID-12SR2: ATCCTTTAATCAGGCACCTA | 48 °C | Designed |

**Table :**
