# Supplementary material for: Complete mitogenome of Olidiana ritcheriina (Hemiptera: Cicadellidae) and phylogeny of Cicadellidae
Source: PeerJ. 2019 Nov 26;7:e8072. doi: 10.7717/peerj.8072 (PMC6883956; doi:10.7717/peerj.8072)
Supplement: Table S2 [file peerj-07-8072-s004.docx]

| **Dataset** | **Subset** | **Best Model** | **Site (bp)** | **Partition names** |
| --- | --- | --- | --- | --- |
| P12  8 partitions  P123  11 partitions  P12-rR  10 partitions | 1 | GTR+I+G | 698 | atp6_pos1, atp8_pos1, cox2_pos1, cox3_pos1 |
|  | 2 | TVM+I+G | 1209 | cox2_pos2, atp6_pos2, cox1_pos2, cox3_pos2, atp8_pos2 |
|  | 3 | GTR+I+G | 888 | cob_pos1, cox1_pos1 |
|  | 4 | TVM+I+G | 377 | cob_pos2 |
|  | 5 | GTR+I+G | 1273 | nad5_pos1, nad4l_pos1, nad1_pos1, nad4_pos1 |
|  | 6 | GTR+I+G | 1273 | nad4l_pos2, nad4_pos2, nad5_pos2, nad1_pos2 |
|  | 7 | GTR+I+G | 513 | nad6_pos1, nad3_pos1, nad2_pos1 |
|  | 8 | GTR+I+G | 513 | nad2_pos2, nad6_pos2, nad3_pos2 |
|  | 1 | GTR+I+G | 741 | nad6_pos1, atp6_pos1, nad3_pos1, nad2_pos1, atp8_pos3, atp8_pos1 |
|  | 2 | TVM+I+G | 1209 | atp6_pos2, cox2_pos2, cox1_pos2, cox3_pos2, atp8_pos2 |
|  | 3 | GTR+I+G | 1814 | cox1_pos3, cox2_pos3, nad6_pos3, nad3_pos3, cob_pos3, atp6_pos3, cox3_pos3 |
|  | 4 | GTR+I+G | 888 | cox1_pos1, cob_pos1 |
|  | 5 | GTR+I+G | 483 | cox3_pos1, cox2_pos1 |
|  | 6 | TVM+I+G | 377 | cob_pos2 |
|  | 7 | GTR+I+G | 1273 | nad4l_pos1, nad5_pos1, nad4_pos1, nad1_pos1 |
|  | 8 | GTR+I+G | 1273 | nad4l_pos2, nad4_pos2, nad1_pos2, nad5_pos2 |
|  | 9 | GTR+I+G | 1273 | nad5_pos3, nad4l_pos3, nad4_pos3, nad1_pos3 |
|  | 10 | GTR+I+G | 513 | nad2_pos2, nad6_pos2, nad3_pos2 |
|  | 11 | TRN+G | 272 | nad2_pos3 |
|  | 1 | GTR+I+G | 698 | atp6_pos1, atp8_pos1, cox3_pos1, cox2_pos1 |
|  | 2 | TVM+I+G | 1209 | cox2_pos2, atp6_pos2, cox1_pos2, cox3_pos2, atp8_pos2 |
|  | 3 | GTR+I+G | 511 | cox1_pos1 |
|  | 4 | GTR+I+G | 377 | cob_pos1 |
|  | 5 | TVM+I+G | 377 | cob_pos2 |
|  | 6 | GTR+I+G | 1273 | nad5_pos1, nad4l_pos1, nad1_pos1, nad4_pos1 |
|  | 7 | GTR+I+G | 1273 | nad4l_pos2, nad4_pos2, nad1_pos2, nad5_pos2 |
|  | 8 | GTR+I+G | 513 | nad6_pos1, nad2_pos1, nad3_pos1 |
|  | 9 | GTR+I+G | 513 | nad2_pos2, nad6_pos2, nad3_pos2 |
|  | 10 | GTR+I+G | 1818 | rrnL, rrnS |
| P123-rR  12 partitions | 1 | GTR+I+G | 741 | nad6_pos1, atp6_pos1, nad3_pos1, nad2_pos1, atp8_pos3, atp8_pos1 |
|  | 2 | TVM+I+G | 1209 | atp6_pos2, cox2_pos2, cox1_pos2, cox3_pos2, atp8_pos2 |
|  | 3 | GTR+I+G | 1814 | cox1_pos3, cox2_pos3, nad6_pos3, nad3_pos3, cob_pos3, atp6_pos3, cox3_pos3 |
|  | 4 | GTR+I+G | 888 | cox1_pos1, cob_pos1 |
|  | 5 | GTR+I+G | 483 | cox3_pos1, cox2_pos1 |
|  | 6 | TVM+I+G | 377 | cob_pos2 |
|  | 7 | GTR+I+G | 1273 | nad4l_pos1, nad5_pos1, nad4_pos1, nad1_pos1 |
|  | 8 | GTR+I+G | 1273 | nad4l_pos2, nad4_pos2, nad1_pos2, nad5_pos2 |
|  | 9 | GTR+I+G | 1273 | nad5_pos3, nad4l_pos3, nad4_pos3, nad1_pos3 |
|  | 10 | GTR+I+G | 513 | nad2_pos2, nad6_pos2, nad3_pos2 |
|  | 11 | TRN+G | 272 | nad2_pos3 |
|  | 12 | GTR+I+G | 1818 | rrnS, rrnL |
| AA  4 partitions | 1 | MTART+I+G+F | 973 | atp6, cox3, nad2, nad3, nad6 |
|  | 2 | MTART+I+G+F | 390 | cob, atp8 |
|  | 3 | MTART+I+G+F | 735 | cox1, cox2 |
|  | 4 | MTART+I+G+F | 1273 | nad4, nad4l, nad1, nad5 |
